# *ETV6*: A Candidate Gene for Predisposition to “Blend Pedigrees”? A Case Report from the NEXT-Famly Clinical Trial

**DOI:** 10.1155/2020/2795656

**Published:** 2020-01-11

**Authors:** Simona Bernardi, Mirko Farina, Camilla Zanaglio, Federica Cattina, Nicola Polverelli, Francesca Schieppati, Federica Re, Chiara Foroni, Michele Malagola, Andrew J. Dunbar, Domenico Russo

**Affiliations:** ^1^Chair of Hematology, Unit of Blood Diseases and Stem Cell Transplantation, Department of Clinical and Experimental Sciences, University of Brescia, ASST Spedali Civili di Brescia, 25123 Brescia, Italy; ^2^CREA Laboratory (Centro di Ricerca Emato-Oncologica AIL), ASST Spedali Civili di Brescia, 25123 Brescia, Italy; ^3^Unit of Transfusion Medicine, ASST Papa Giovanni XXIII, 24127 Bergamo, Italy; ^4^Department of Medicine, Leukemia Service, Memorial Sloan Kettering Cancer Center, New York, NY, USA

## Abstract

**Background:**

The identification of germline mutations in familial leukemia predisposition genes by next generation sequencing is of pivotal importance. Lately, some “blend pedigrees” characterized by both solid and hematologic malignancies have been described. Some genes were recognized as related to this double predisposition, while the involvement of others is still a matter of debate. *ETV6* was associated with hematologic malignancies, in particular myeloid malignancies, and recently described as mutated also in oncologic patients. No clear evidences in its involvement in blend pedigrees are known. *Case Presentation*. We present our recent experience in the identification of an *ETV6* was associated with hematologic malignancies, in particular myeloid malignancies, and recently described as mutated also in oncologic patients. No clear evidences in its involvement in blend pedigrees are known. *ETV6* was associated with hematologic malignancies, in particular myeloid malignancies, and recently described as mutated also in oncologic patients. No clear evidences in its involvement in blend pedigrees are known. *ETV6* was associated with hematologic malignancies, in particular myeloid malignancies, and recently described as mutated also in oncologic patients. No clear evidences in its involvement in blend pedigrees are known.

**Conclusion:**

This evidence supports the involvement of *ETV6* in the predisposition to both solid and hematologic neoplasia and the importance of the investigation of the noncoding regions of the genes as recently suggested by different expert groups.*ETV6* was associated with hematologic malignancies, in particular myeloid malignancies, and recently described as mutated also in oncologic patients. No clear evidences in its involvement in blend pedigrees are known.

## 1. Introduction

Acute myeloid leukemias (AML) and myelodysplastic syndromes (MDS) are clonal disorders of hematopoietic stem cells. With the advent of next-generation sequencing (NGS) technology, significant progress has been made in furthering our understanding of the molecular pathophysiology and complexity of these diseases [1]. NGS has also led to an increasing clinical awareness of inheritable leukemia syndromes and the genes involved. It is well established that several inheritable single-gene syndromes are associated with AML and MDS, including bone marrow failure syndromes (BMFS), Li-Fraumeni syndrome, and Down syndrome [2], which tend to affect individuals at a younger age. Widespread incorporation of a detailed medical and family history into the clinical care of every patient with unexplained cytopenias, aplastic anemia, or MDS/AML will continue to identify an increasing number of individuals with these disorders.

Family pedigrees of inheritable MDS/AML have been previously reported [3–5]. Detailed genetic investigations of these pedigrees have led to the identification of a number of individual genes that predispose to the development of AML and/or MDS [6]. Other data suggest these genes, when comutated with acquired somatic alterations, ultimately lead to the development of overt myeloid disease. The identification of additional potential candidate genes have been recently published in limited reports, including *SRP72* [7].

The clinical relevance of these syndromes is highlighted by the updated World Health Organization (WHO) classification of myeloid neoplasia, which now recognizes “Myeloid Neoplasm with Germline Predisposition” as a separate entity [8]. These syndromes are often associated with inherited mutations often affecting genes critical to hematopoiesis [9], including *RUNX1, CEBPα*, and *ETV6*. The WHO (Table 1) classifies three general categories of germline myeloid neoplasms: “pure” familial MDS and AML with no significant nonhematopoietic pathology, MDS/AML-susceptibility mutations with concomitant platelet abnormalities, and MDS/AML occurring in the setting of an underlying clinical genetic syndrome, including those characterized by BMFS (Table 2). For all these disorders, MDS and AML are often the predominant hematopoietic malignancies; however, lymphoid neoplasia as well as solid tumors can also occur [10–14].

NGS has been essential to the identification of novel molecular alterations in familial MDS/AML, and it has contributed to further understanding of inherited leukemia syndromes, and improved identification of inherited AML predisposition alleles. This has opened the opportunity to carry out a personalized management approach to affected patients [15–17]. For example, because long-term outcomes of MDS secondary to BMFS and familial MDS/AML are historically poor, hematopoietic stem cell transplantation should be considered sooner in the disease course, with exception for patients with germline mutations in *CEBPα* [18]. Nevertheless, a diagnosis of familial MDS/AML adds several considerations to pretransplant planning, namely, in the choice of the conditioning regimen and the selection of the hematopoietic stem cell donor [19–21].

The cases of MDS/AML with germline predisposition also differ from typical sporadic MDS and AML in that they are frequently associated with unique nonhematopoietic manifestations, requiring more dedicated disease surveillance [18].

In order to better recognize patients who may have an inherited predisposition to leukemia and MDS, clinicians must take a complete family history and be familiar with the characteristics of familial MDS/AML syndromes. In this scenario, evaluation of the family pedigree often reveals multiple family members affected by both hematologic and solid tumors. Moreover, other genes conventionally associated with solid tumors inheritance, such as *BRCA1* and *BRCA2*, have also been more recently been implicated in both pediatric and adult MDS/AML cases [22, 23].

In parallel with these well-established evidences, there is a group of four genes still considered as associated with a “possible tumor risk” that must be confirmed. *PTPN11* is not included in the ones recognized by the WHO classification, while *DDX41, GATA2*, and *ETV*6 have been already linked with defined familial MDS/AML [24].

*ETV6* is located at chromosome 12p13 and encodes a hematopoietic transcription factor involved in early embryonic and adult hematopoiesis as well as in the regulation of differentiation and maturation of erythropoietic and megakaryopoietic cell lineages [25, 26].

Alterations of *ETV6* have been shown to be important players in leukemogenesis since its recognition as a *PDGFB* translocation partner in chronic myelomonocytic leukemia [27]. *ETV6* is also frequently found to be partnered in numerous other variant translocations across hematologic malignancies which affect *ETV6* transcription and gene regulation [28]. Moreover, deletions [29] and somatic mutations in *ETV6* have also been reported in myeloid disease, including in MDS [30–32] and AML [33] as well as in CML [34], and are generally associated with poor outcomes [29].

More recently, germline mutations of *ETV6* have been identified in a small number of family pedigrees. Germline mutations involving the *ETV6* gene are often missense and inherited in an autosomal dominant fashion (Table 3), even if the penetrance of hematologic neoplasia depends both on the different mutated locus and between families affected by the same *ETV6* variant [26]. Patients often have variable thrombocytopenia with mild-to-moderate bleeding tendency and are predisposed to develop both myeloid and lymphoid malignancies. In particular, *ETV6* is suggested to be mostly predisposing to acute lymphoblastic leukemia rather than to myeloid malignancies [35], even if it was not reported in the conventional WHO classification.

Notably, additional nonhematological malignancies in *ETV6*-mutant carriers have also been documented [36–41]. With the paucity of germline-mutated *ETV6* cases published, additional mutations are still being discovered. Given this, we describe herein a novel mutation of *ETV6* found in an individual family pedigree linked to both hematologic and solid tumor malignancy.

## 2. Case Presentation

**Figure 1:**
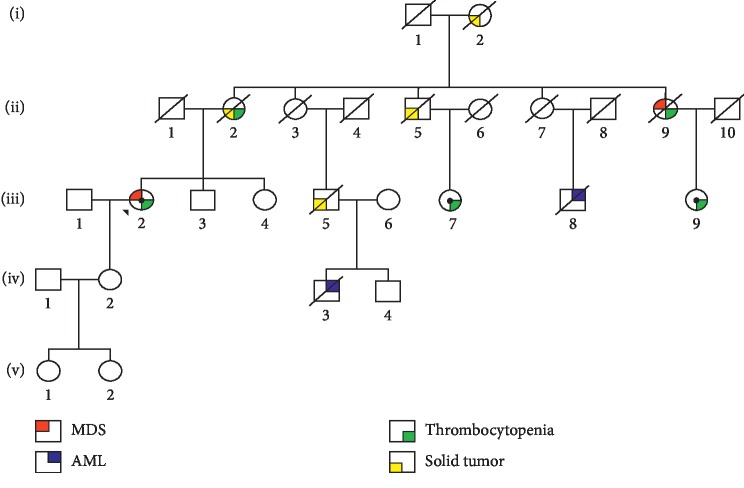
Case index pedigree (black arrow indicates the case index; black dot indicates subject presenting mutation; MDS = myelodisplastic syndrome; AML = acute myeloid leukemia).

We analyzed a 70-year-old woman who presented with progressive thrombocytopenia that has been present for 8 years, and monocytosis in June 2014. The bone marrow biopsy performed at that time showed a MDS with multilineage dysplasia according to WHO classification [8], with a bone marrow blast count percentage totaled <5%. Cytogenetics revealed a translocation (3; 21). Therefore the patient falls within the intermediate 1 risk group, according to IPSS. She was started on danazole 400 mg/day which was eventually increased to 600 mg/day, with a low response in platelets count (30–40,000/mmc). The treatment was well tolerated, and no major bleeding was observed. After two years on therapy, she again developed worsening thrombocytopenia (10,000/mmc). Repeat bone marrow evaluation revealed MDS with excess blasts type 1 (MDS-EB 1). She was subsequently treated with 5-azacitidine at a dose 75 mg (sqm)/day x 7 days monthly for 15 cycles, for which she achieved a complete hematological response after 4 cycles. The treatment was discontinued in November 2017 for hematological toxicity and evolution of the disease to MDS-EB type 2 (IPSS intermediate 2). The patient subsequently evolved to AML and died due to disease progression. Her family history revealed several cases of hematological disease (2 cases of AML diagnosed at 62 and 48 years, 1 case of MDS diagnosed at 60 years associated with history of thrombocytopenia, and 2 cases of thrombocytopenia diagnosed at 49 and 57 years). In addition, several solid neoplasms distributed over 4 generations were also present (2 cases of gastric cancer diagnosed at 58 and 42 years, 1 pulmonary adenocarcinoma associated with history of thrombocytopenia diagnosed at 52 years, and 1 undetermined) (see Figure 1). Due to these evidences, the patient provided written informed consent in accordance with the Declaration of Helsinki and she was enrolled in the Next-Famly Italian Multicentric Study (NCT03058588). The written informed consent was attached to the patient's medical record as recommended by the Good Glinical Practice (ICH) guidelines [42]. As part of the study, a gene panel deep sequencing (GPDS) was performed on isolated peripheral blood mononuclear cells (PBMCs) at time of diagnosis. Tumor DNA was first screened by using the MiSeq Illumina NGS platform for mutations in the following 25 genes: *ASXL1, BCOR, NRAS, TP53, RUNX1, CEBPA, FLT3, EZH2, IDH1, IDH2, NPM1, DNMT3A, TET2, CBL, KRAS, ETV6, SF3B1, SRSF2, U2AF1, ZRSR2, GATA2, TERT, TERC, SRP72,* and *ANKRD26*. The sequencing focused only on exons and/or regulatory domains of each gene. The library preparation was conducted following the manufacturer's instructions, and DNAs were sequenced by MiSeq Illumina NGS platform using 2 × 150 sequencing (V2 kit, TruSeq). The coverage considered as acceptable for the consistence of the results was fixed at 1500x. The data analysis was performed using Web Annovar. During bioinformatics analysis, polymorphisms were discarded by comparison with NCBI, dbSNP, 1000 genomes, and EXAC, automatically investigated by using Web Annovar. However, since these databases contain known disease-associated mutations, all detected variants were compared with gene-specific mutation databases, ClinVar, and COSMIC. The ranking of unknown mutations was performed using Sift, Polyphen2, Mutation tester, FATHMM, ProVean, MetaSVM, M-CAP.

Review of NGS in this patient revealed a novel point mutation in *ETV6* at c.514 C>*T* in the 3′UTR locus not present in the interrogated database.

Since a suspected mutation was found, germline DNA from epithelial buccal cells was sequenced using traditional Sanger methods (SS). Germline DNA was extracted from buccal epithelial cells collected using the Isohelix SK-2 DNA Buccal Swab Collection Kit [43].

**Figure 2:**
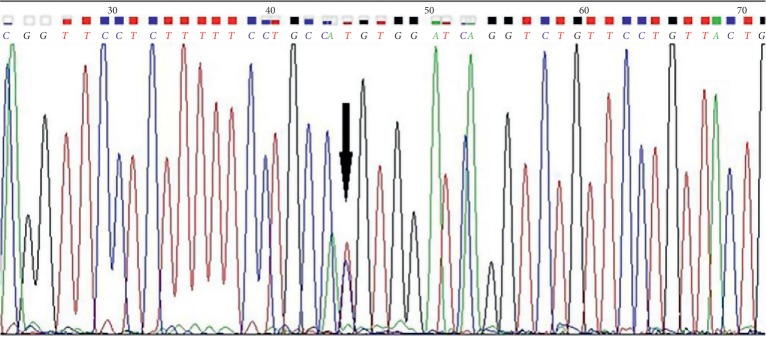
Electropherogram of the locus containing the mutation revealed by Sanger sequencing. The black arrow indicates the point mutation.

SS confirmed the presence of the variant c.514C>*T* of *ETV6* on the germline DNA of the index case (Figure 2). Considering the presence of a germline mutation, affected relatives still living at time of analysis were enrolled in the study and tested for the presence of the identified mutation. The germline mutation was confirmed in two enrolled cousins. Unfortunately, DNA was not available for additional testing on family members affected by solid tumors as they had already deceased.

**Figure 3:**
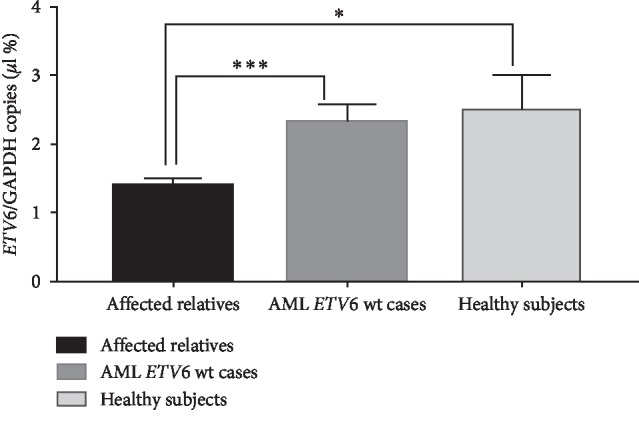
Quantification of *ETV6* transcripts by dPCR. The quantification was performed on PBMCs RNA. *ETV6* transcript levels were normalized for *GAPDH* transcript levels and expressed as ratio. Black block: affected relatives; Dark grey block: AML wt-*ETV6* cases; Light grey block: healthy subjects. Affected relatives presenting ^*∗*^514C>*T* variants resulted with *ETV6* statistically down regulated in comparison with AML cases presenting wild-type *ETV6* (^*∗∗∗*^*P*=0.0004) and healthy controls (^*∗*^*P*=0.02). PBMCs = peripheral blood mononuclear cells; AML = acute myeloid leukemia.

miRANDA (microRNA.org) and PolymiRST Database Version 3.0 analysis suggested that the affected *ETV6* 3′UTR sequence serves as an miRNA-binding site, specifically the regulatory microRNA hsa-miR5092. Notably, the mutated one was predicted to bind microRNAs, hsa-miR4717-3p and hsa-miR942-3p. microRNAs have a known role in gene regulation. In order to assess the potential effect of the mutation on *ETV6* transcription in the index case, *ETV6* mRNA was isolated from PBMCs and quantified on the QuantStudio 3D digital PCR system (ThermoFisher Scientific) using the Hs01045747_m1 Taqman Gene probe (ThermoFisher Scientific) with Hs039290997_g1 Taqman *GAPDH* probe (ThermoFisher Scientific) as a housekeeper gene. Ten AML cases presenting with wild-type *ETV6* as well as 3 healthy individuals served as controls. The results of the quantification of *ETV6*, normalized for *GAPDH* transcript, are reported in Figure 3. Notably, affected relatives as well as the index case harboring the ^*∗*^514C>*T* mutation demonstrated a significant down regulation of *ETV6* gene expression in comparison to AML *ETV6* wild-type (*P*=0.0004) and healthy controls (*P*=0.02).

## 3. Discussion

The identification of some genes involved in inheritance of hematological malignancies allowed to recognition of an independent entity in last revision of WHO classification and also represents a further step toward the precision and personalized therapy [15, 24].

Moreover, the study of some pedigrees highlights the possible involvement of some genes in both tumorigenesis and leukemogenesis [44]. The definition of intermediate phenotype typically defined as “blend pedigree” leading to a high prevalence of both solid and hematological neoplasia is mandatory to clarify both the biological processes involved in the development of these different malignancies and for the establishment of an efficient treatment strategy and clinical survival.

Some “blend pedigrees” have already been reported in the literature and some genes have been linked to this condition. The implication of some of them in the pathogenesis of genetic predisposition to neoplasia is still a matter of debate. Recent evidences highlighted the importance of sequencing by NGS in these patients and also of investigating the regulatory and intragenic portions instead of the only coding regions which are conventionally queried [17, 24]. This approach is still mandatory for the study of *ANKRD26* since the known mutations associated with MDS/AML pedigrees affect the 5′UTR of the gene [45].

The family presented in this paper has been recognized thanks to the investigation of the noncoding sequences of all the genes included in our panel. The mutation was detected in 3′UTR of *ETV6* and no variants in this locus has been described before as causative of MDS/AML familiarity.

*ETV6* is conventionally associated to hematological diseases both of lymphoid and myeloid lineages [35, 45], but more recently it has been implicated in both liquid and solid tumor malignancies (so-called “blend families”). The lack of acute lymphoblastic leukemia cases in the described family is uncommon and it might be due to the mutated locus, never described before. The mutation is associated with a downregulation of *ETV6* transcript, while the previously reported mutations modify the *ETV6* protein structure. This different pathogenetic mechanism may affect lesser the lymphoid lineage than the myeloid one. Moreover, *ETV6* ^*∗*^514C>*T* seems to be associated with double predisposition to both hematologic and solid tumor malignancies and not to the conventional predisposition to hematologic disorder described in the literature [8, 37, 40, 41]. The pedigree we studied and reported was strongly suspected to be a “blend pedigree” in that the *ETV6* mutation–identified mutation might have led to both hematological and solid tumor malignancies. Recent studies reinforce this hypothesis since *ETV6* has been reported as a tumor suppressor also in primary cutaneous mammary analog secretory carcinoma [46], of pediatric papillary thyroid cancers [47], and in gastric cancer [48]. In particular, this last paper is particularly interesting due to the presence of 2 cases of gastric cancers in the described family. Unfortunately, due to the lack of the deceased patients' permission for further investigation, it was not possible to analyze the presence of the *ETV6* mutation in relatives affected by solid tumors.

Another important perspective that could be investigated in the future is the relationship between the t(3; 21), detected in the reported case, and *ETV6* 3′ UTR mutations. A possible cooperation of *ETV6 *^*∗*^514C>*T* with this or other lesions may be evaluated. In fact, in absence of WGS analysis, we cannot be sure that ^*∗*^514C>*T* in *ETV6* is the only causative mutation of the high susceptibility of the described family's members, but considering the evidences in the scientific literature, the reduction of the *ETV6* expression and the presence of the mutation in all alive affected family members, we may assess that the described mutation is strongly suspected to predispose to hematologic and solid malignancies. Nevertheless, further investigation on cell and animal models will give us pivotal data among the leukemogenesis process, since no evidences concerning this aspect have been reported.

Since the role of this variant in leukemogenesis and tumorigenesis has to be confirmed, we suggest to always carry out a careful family history because many pedigrees, strongly suspected to be “blend pedigree,” could be misunderstood.

Our experience adds an indication of a possible involvement of *ETV6* in the “blend pedigrees” field, but new studies have to be conducted to investigate familiar predisposition to malignancies with a special focus on these entities, which are still an unexplored scenario.

## Figures and Tables

**Table 1:** WHO-defined myeloid neoplasm with germline predisposition.

Myeloid neoplasm with germline predisposition without preexisting disorder or organ dysfunction
(i) *CEBPA* mutation
(ii) *DDX41* mutation

*Myeloid neoplasm with germline predisposition and preexisting platelet disorders*
(i) *RUNX1* mutation
(ii) *ANKRD26* mutation
(iii) *ETV6* mutation

*Myeloid neoplasm with germline predisposition and other organ dysfunction*
(i) *GATA2* mutations
(ii) Telomere biology disorders
(iii) Bone marrow failure syndrome (Fanconi anemia, dyskeratosis congenita, severe congenital neutropenia, Swachman–Diamond syndrome, and Blackan-Diamond syndrome)

**Table 2:** Characteristics of AML/MDS predisposition syndromes.

Disease	Clinical characteristics	Mutated gene	Pattern of inheritance	Penetrance
Familial AML with mutated *CEBPA*	AML	*CEBPA*	AD	≈100%
Myeloid neoplasm with germline *DDX41* mutation	MDS/AML	*DDX41*	AD	Unknown
Familial platelet disorder/AML	MDS/AML/T cell ALL, lifelong thrombocytopenia, bleeding propensity	*RUNX1*	AD	40%
Thrombocytopenia and predisposition to myeloid malignancies	Thrombocytopenia, platelet dysfunction, MDS/AML	*ANKRD26*	AD	Unknown
Myeloid neoplasm with germline *ETV6* mutation	Thrombocytopenia, platelet dysfunction, MDS/AML	*ETV6*	AD	≈100%
Familial MDS/AML with mutated *GATA2*	MDS/AML, MonoMAC syndrome, Emberger syndrome	*GATA2*	AD	70%
Telomere biology disorders	MDS/AML, macrocytosis, mild to moderate single or multiple cytopenias, aplastic anemia	*TERT, TERC*	AD	Variable
Bone marrow failure associated with *SRP72* mutations	Aplastic anemia, MDS	*SRP72*	AD	Unknown

AML = acute myeloid leukemia; MDS = myelodisplastic syndrome; AD = autosomic dominant.

**Table 3:** Main hotspot mutations in the *ETV6* gene and their impact on the protein.

Mutation	Domain	Effect
P214L	Central regulatory domain	(i) Repression of DNA binding by the ETS domain (ii) Defective proplatelet formation and megakaryocyte maturation (iii) Alteration of proplatelet spreading (iv) Down regulation of several cytoskeletal proteins (v) *ETV6* delocalization

N385Vfs	ETS	(i) Reduction in repressive activity (ii) Targeted proteins downregulation

Y401N	ETS	(i) Impaired interaction with corepressor (ii) Defective proplatelet formation and megakaryocyte maturation

R369W/R369Q	ETS	(i) Reduction in repressive activity (ii) Targeted protein downregulation (iii) *ETV6* delocalization

ETS = highly conserved C-teminal DNA-binding domain.

## Abbreviations

AML: Acute myeloid leukemia
MDS: Myelodysplastic syndrome
NGS: Next-generation sequencing
BMFS: Bone marrow failure syndromes
WHO: World Health Oganization
IPSS: International Prognostic Scoring System
MDS-EB: Myelodysplastic syndrome with excess of blasts
SS: Sanger sequencing
GPDS: Gene panel deep sequencing.
